# O Uso da Escala de Borg na Percepção do Esforço em Manobras de Reanimação Cardiopulmonar

**DOI:** 10.36660/abc.20220240

**Published:** 2022-12-20

**Authors:** Lucia Tobase, Heloisa Helena Ciqueto Peres, Thatiane Facholi Polastri, Sandra Helena Cardoso, Dhieizom Rodrigo Souza, Debora Gugelmin Almeida, Sergio Timerman

**Affiliations:** 1 Centro Universitário São Camilo São Paulo SP Brasil Centro Universitário São Camilo, São Paulo, SP – Brasil; 2 Escola de Enfermagem Universidade de São Paulo Departamento de Orientação Profissional São Paulo SP Brasil Escola de Enfermagem da Universidade de São Paulo – Departamento de Orientação Profissional, São Paulo, SP – Brasil; 3 Universidade de São Paulo Instituto do Coração São Paulo SP Brasil Universidade de São Paulo Instituto do Coração, São Paulo, SP – Brasil

**Keywords:** Parada Cardíaca, Esforço Físico, Reanimação Cardiopulmonar, Cuidados de Enfermagem, Emergências

## Abstract

**Fundamento:**

A parada cardiorrespiratória é um evento crítico cuja taxa de sobrevivência é relacionada à qualidade das manobras de reanimação, aliada à tecnologia. É importante compreender a percepção do cansaço durante esse procedimento visando a efetividade das compressões e o aumento das chances na sobrevida.

**Objetivo:**

Aplicar a Escala de Borg para analisar o esforço percebido por enfermeiros durante as manobras de reanimação cardiopulmonar com dispositivo de feedback.

**Método:**

Estudo experimental com distribuição randomizada de enfermeiros em hospital de ensino, simulando parada cardiorrespiratória, para avaliação da percepção do esforço utilizando a escala de Borg durante a reanimação cardiopulmonar com/sem dispositivo de feedback. Foi adotado nível de significância estatística 5%.

**Resultados:**

Foram incluídos 69 enfermeiros atuantes em unidades críticas e não críticas de atendimento ao adulto. A percepção de esforço e a frequência cardíaca foi menor no grupo intervenção (p<0,001), influenciadas pelo dispositivo de feedback, sem diferença significativa quanto às unidades de atuação.

**Conclusão:**

A escala de Borg mostrou-se adequada para os objetivos propostos. O dispositivo de feedback contribuiu no menor esforço e redução da frequência cardíaca durante as manobras de reanimação. O baixo custo e a facilidade de aplicação favorecem o uso em treinamentos e atendimentos em tempo real para avaliar o desempenho durante a reanimação, utilizando dispositivo de feedback por reduzir os esforços e a percepção do cansaço. Também permite a reflexão sobre os fatores intervenientes e recursos que podem influenciar na qualidade da assistência e nas chances de sobrevivência.

## Introdução

No panorama mundial, a parada cardiorrespiratória (PCR) é responsável por cerca de 17,8 milhões de mortes/ano.^1^ Apesar dos avanços na ciência da ressuscitação, desde 2012, a sobrevivência ainda permanece em cerca de 8-10%. A compreensão da epidemiologia ainda é limitada pela carência de dados globais, regionais e registros fidedignos do evento, particularmente em países de baixa e média renda. As diretrizes da *American Heart Association* (AHA) de 2020 destacam a reanimação cardiopulmonar (RCP) de alta qualidade como fator de sucesso na intervenção e maior taxa de sobrevivência.^2^

A qualidade da realização da RCP também depende da condição física do socorrista, cansaço e fadiga são fatores que comprometem a sobrevivência. O revezamento entre os socorristas a cada 2 minutos visa evitar fadiga e má qualidade das manobras – comuns após 1 minuto de RCP – não identificadas mesmo após 5 minutos ou mais, do início da intervenção.^2 , 3^

Para mensurar a intensidade da atividade física é aplicada a escala de esforço percebido ou classificação do esforço percebido (PSE) ou escala de Borg. É uma ferramenta eficaz para prever o desempenho e definir estratégias para aumentar a qualidade da atividade física.^4^ A mensuração dos níveis de recuperação também pode ser analisada pela escala de Borg^5^após esforço físico. No contexto da RCP, é utilizada para avaliar a qualidade do desempenho e as chances de melhorar as tentativas de ressuscitação.^4^

No suporte à RCP, o uso dos dispositivos de *feedback* são encorajados em atendimento real ou treinamento.^2^ Apesar das evidências claras de que oferecer RCP de alta qualidade melhora os resultados da ressuscitação, poucas organizações de saúde aplicam estratégias consistentes no monitoramento da qualidade da RCP. Consequentemente há uma disparidade inaceitável na qualidade dos cuidados e resultados de ressuscitação, diante da enorme oportunidade para salvar mais vidas.^6^

O presente estudo tem por hipótese que há diferenças na percepção do esforço, ao prover o Suporte Básico de Vida, com ou sem dispositivos de *feedback* imediato. E tem por objetivo aplicar a Escala de Borg para analisar o esforço percebido por enfermeiros durante as manobras de reanimação cardiopulmonar com dispositivo de feedback.

## Métodos

### Delineamento do estudo

Estudo de natureza quantitativa, do tipo experimental, para comparar a influência do dispositivo de *feedback* na percepção do esforço durante a reanimação realizada por enfermeiros, distribuídos aleatoriamente em grupo controle e intervenção, no período de outubro a novembro de 2020. O duplo cegamento foi inviabilizado, visto que o instrumento escala de Borg foi aplicado pela própria pesquisadora, na avaliação das variáveis percepção do esforço, e da FC aferida com frequencímetro, considerado sensor preciso que fornece medições de boa qualidade. Previamente ao uso da escala foi efetuado contato com os responsáveis (https://borgperception.se/) para esclarecimentos sobre a pesquisa e solicitação da anuência para seu uso, o qual foi autorizado. A pesquisa foi aprovada pelo Comitê de Ética em Pesquisa.

### Local do estudo

Hospital de ensino, público, geral, de médio porte, do tipo secundário e média complexidade, localizado na cidade de São Paulo, SP, Brasil.

### Participantes do estudo

Enfermeiros atuantes em unidades de atendimento ao adulto, críticas (Unidade de Terapia Intensiva, Pronto Socorro, Centro Cirúrgico e Centro Obstétrico) e não críticas (Clínica Médica, Clínica Cirúrgica e Ambulatório).

### Critérios de inclusão e exclusão

Foram incluídos os enfermeiros assistenciais de unidades de atendimento ao adulto e excluídos os que atuavam apenas em atividade administrativa ou como instrutores em cursos de Suporte Básico ou Avançado de Vida. Em atenção à segurança, os profissionais impossibilitados de realizar ou concluir a atividade na integralidade, por limitação física ou gestação, ou que apresentavam sintomas de dor ou problemas de saúde também foram excluídos.

### Delineamento do percurso metodológico

1. Apresentação da pesquisa aos gestores do serviço.
2. Recrutamento dos enfermeiros e envio, via *e-mail,* das informações da pesquisa e obtenção do consentimento.
3. Agendamento e realização da atividade prática, em duas etapas: 1ª) *Baseline* : verificação das habilidades em suporte básico de vida. Para alinhamento teórico e atualização das diretrizes AHA/2020 os participantes acessaram curso *online* desenvolvido pela pesquisadora. 2ª) Após o estudo teórico, os enfermeiros participaram de segunda atividade prática, nos moldes da primeira.

Os participantes foram alocados em dois grupos, intervenção e controle, conforme listagem de distribuição aleatória gerada em computador pelo estatístico. Respectivamente simularam atendimento em PCR e manobras de Suporte Básico de Vida, com e sem dispositivo de *feedback* . Após orientações e *briefing* sobre a atividade e os recursos disponíveis, na apresentação e ambiência com o cenário e os instrumentos a serem utilizados, o frequencímetro Polar H10^®^ foi colocado em cada enfermeiro para medir a frequência cardíaca (FC). Os registros armazenados na memória interna do equipamento eram transferidos via *Bluetooth* a telefone celular e *tablet* ; posteriormente foram acessados no *site* (https://flowpolar.com) e tabulados em planilha *Excel* ^®^ para gestão das informações.

Sobre a dinâmica da simulação RCP: a) apresentação do caso clínico; b) enfermeiro 1 identifica a PCR e inicia as compressões torácicas; c) enfermeiro 2 assume as ventilações e usa o desfibrilador manual no modo DEA, durante 2 minutos; d) ao final, pausa e descanso, por cerca de 10 minutos, para higienização de mãos e materiais, e reinício da atividade, com inversão dos papeis entre os profissionais. A escala de Borg foi aplicada quando o enfermeiro realizava as compressões. Cada atendimento foi acompanhado por dois avaliadores. Foram utilizados manequim simulador *Little Anne QCPR* ^®^ com dispositivo de *feedback* visualizável em *smartphone* ( *QCPR instructor app* ) e carro de emergência disponibilizado pelo serviço com prancha rígida, bolsa-válvula-máscara, fluxômetro com extensão e desfibrilador manual no modo DEA – modelo Desfibrilador Bifásico *Zoll M Series* ^®^.

A escala de Borg foi previamente apresentada aos participantes para esclarecimentos dos critérios e familiarização com o instrumento. Ao longo da atividade prática foi aplicada no primeiro e segundo minuto durante a realização das compressões e após o atendimento, em pausa para avaliar a recuperação do cansaço. Os valores de FC no frequencímetro foram registrados. Os valores da escala de Borg (6-20) utilizada na presente pesquisa variaram entre: 6-11 representa o esforço mínimo, 12-16 para o esforço sustentável e 16-20 quanto ao esforço não sustentável até a exaustão.

### Análises estatísticas

Nas análises estatísticas descritivas e inferenciais utilizou-se o *software R* ^®^ 4.1.0 adotando nível de significância 5%. As estatísticas descritivas foram utilizadas para explorar os dados demográficos. As variáveis de interesse em relação ao desfecho dizem respeito ao escore na percepção de esforço da escala de Borg e variação da frequência cardíaca, durante a RCP. As variáveis categóricas incluíram atuação dos participantes em unidades críticas e não críticas e foram descritas em frequências relativas e absolutas. Foram utilizados testes Skewness, Kurtosis e Shapiro-Wilk para determinar a normalidade. As variáveis contínuas apresentaram distribuição normal, sendo descritas através de média e desvio padrão (DP). Utilizou-se modelo de efeitos mistos para comparar as variáveis de esforço percebido e FC.

## Resultados

**Figura 1:**
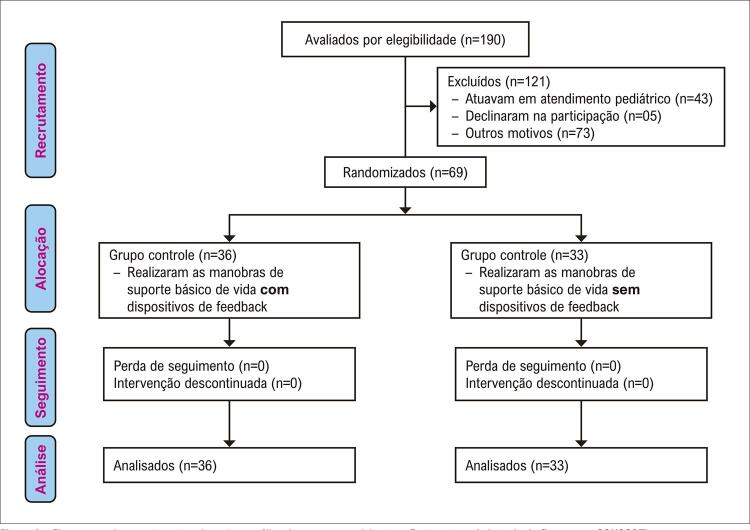
Fluxograma do recrutamento, alocação e análise dos grupos participantes. Fonte: autora (adaptado do fluxograma CONSORT).

Dos 190 enfermeiros na instituição, 72 (38%) foram excluídos por atuarem em áreas não relacionadas ao foco do estudo. Dos 118 (62%) elegíveis, 62 (53%) atuavam em unidades críticas e 56 (47%) em unidades não críticas. Destes, 49 (41%) foram excluídos por problemas de saúde, licença médica, trabalho remoto devido à pandemia por COVID-19 e desligamento da instituição, conforme Figura 1 .

Foram incluídos 69 enfermeiros, 35 (51%) de unidade crítica e 34 (49%) de unidade não crítica; 3 (04%) concluíram doutorado, 15 (22%) mestrado, 44 (64%) especialização e 7 (10%) sem titulação pós-graduação. Demais caracterizações do perfil dos participantes conforme Tabela 1 .

**Tabela 1:** Perfil dos participantes, distribuídos entre grupo controle e intervenção

Variáveis	Intervenção	Controle
	
	n=36 (52%)	n= 33 (48%)
		
Sexo	04 (11%) homens	03 (09%) homens
	32 (89%) mulheres	30 (91%) mulheres
Idade (média em anos/desvio padrão)	41,6 (10,64)	41,4 (10,16)
Índice massa corporal (média/desvio padrão)	25,7 (4,38)	25,6 (4,25)
Unidade crítica	20 (55%)	15 (45%)
Unidade não crítica	16 (44%)	18 (55%)
Formação profissional (média em anos/desvio padrão)	17,6 (10,31)	17,5 (9,95)
**Tempo de atuação do profissional:**		
Na instituição (média em anos/desvio padrão)	15,7 (10,02)	15,5 (9,88)
Na unidade (média em anos/desvio padrão)	12,6 (9,25)	12,5 (9,27)
Prática de atividade física regular	15 (41,6%)	11 (33,3%)

Na segunda etapa, indicada como tempo pós, as descrições quantitativas dos valores são relacionadas às medidas verificadas por meio da escala de Borg e do frequencímetro durante os dois minutos de RCP e no período de recuperação, em pausa para descanso e recuperação do esforço, ao realizar as manobras de SBV, nos grupos intervenção e controle. Na percepção do esforço verificou-se que, inicialmente, a média 6 indicou ausência do cansaço ao iniciar a atividade prática. Progressivamente, ao final do primeiro minuto de RCP, equivalente a cinco ciclos de 30 compressões alternadas com 2 ventilações, o escore entre 13-14 indicava percepção de cansaço moderado, no esforço tolerável para a atividade. Ao final do segundo minuto, após ter totalizado cerca de 10 ciclos alternando 30 compressões e 2 ventilações, a variação de escore 14 indicava esforço moderado, se aproximando do limite para escore 15 na percepção de alta intensidade e dificuldade para realizar a atividade. Nessa direção, verificou-se o aumento progressivo da FC registrada desde o período inicial da atividade e durante a realização dos ciclos de 30 compressões e 2 ventilações.

Em contraposição, na etapa de recuperação verificou-se o decréscimo dos valores na escala de Borg e da FC, indicando, respectivamente, que após quatro minutos o escore de percepção do esforço realizado se aproximava da medida verificada, ao início da atividade. Similarmente, a recuperação ocorreu na verificação da FC, como medida fisiológica que foi se aproximando dos níveis basais, prévios, conforme Tabela 2 .

**Tabela 2:** Variação do esforço percebido na escala de Borg e da FC no primeiro e segundo minuto de RCP, e nos minutos seguintes na recuperação, em grupo controle e intervenção

			BORG		FC*	
	
Grupo	Momento	n	Média	DP^**†**^	Média	DP
**Controle**	Início	33	6,00	0,00	86,50	10,80
	1 minuto	33	14,00	2,15	121,00	16,00
	2 minutos	33	14,90	2,02	123,00	16,80
	Recuperação 1 minuto	33	11,50	1,44	94,20	16,20
	Recuperação 2 minutos	33	9,21	1,80	82,20	13,50
	Recuperação 3 minutos	33	7,88	1,85	79,80	12,10
	Recuperação 4 minutos	33	6,79	1,32	78,90	12,30
**Intervenção**	Início	36	6,00	0,00	88,00	11,30
	1 minuto	36	13,50	2,23	119,00	17,20
	2 minutos	36	14,20	2,41	121,00	18,00
	Recuperação 1 minuto	36	12,00	2,20	95,60	12,40
	Recuperação 2 minutos	36	9,97	1,98	85,50	12,00
	Recuperação 3 minutos	36	8,46	1,90	83,20	11,60
	Recuperação 4 minutos	36	6,94	1,37	81,60	10,30

*FC: frequência cardíaca. †DP: desvio padrão.

Em relação aos enfermeiros atuantes em unidades críticas e não críticas, ao analisar os escores da escala de Borg no primeiro e segundo minuto de RCP, os valores entre 11-13, se aproximando de 14 na realização das compressões, indicaram o aumento da percepção de esforço leve para moderado, se aproximando do esforço intenso ao final do segundo minuto. No período de recuperação, o decréscimo dos valores ao final do quarto minuto foi similar em ambos os grupos. As médias foram muito similares, sugerindo não haver diferença na percepção do esforço e na variação de FC.

Na análise comparativa entre as variáveis esforço percebido e FC, respectivamente registradas por meio da escala de Borg e do frequencímetro, utilizou-se o modelo de efeitos mistos, que incorpora efeitos fixos e aleatórios simultaneamente. Efeitos fixos são aqueles que não têm variabilidade como alocação em grupo controle/intervenção, sexo ou idade de um sujeito (i.e., a grande maioria das variáveis), enquanto efeitos aleatórios são os sujeitos e a variabilidade na seleção. No contexto de dados longitudinais, é tipicamente o sujeito avaliado, ou quando há vários juízes avaliando um conjunto de observações, sendo que esses juízes foram escolhidos de um grupo maior.

A primeira etapa ou *baseline* correspondeu à fase denominada pré e a segunda etapa, com randomização dos participantes, para uso ou não do dispositivo de *feedback* , equivaleu a fase pós. Para ambas as variáveis, os resultados mostraram evidência de interação momento*grupo, o que indica que os grupos provavelmente não têm o mesmo desenvolvimento do início do procedimento até o fim da recuperação. O grupo controle apresentou aumento na percepção de esforço, na FC e recuperação mais demorada. O grupo intervenção apresentou menor percepção de esforço, da FC máxima e recuperação mais rápida, com diferença significativa (p<0,001) na realização de RCP com dispositivo de *feedback* , conforme Tabela 3 .

**Tabela 3:** Esforço percebido segundo a escala de Borg e variação da FC em relação ao tempo, momento e grupo de alocação dos participantes

	BORG			FC*		
	
	Chisq^**†**^	Df^**‡**^	Pr(>Chisq)^**§**^	Chisq	Df	Pr(>Chisq)
(Intercept)	4595,82	1	< 0,001	5190,58	1	< 0,001
Tempo	12,39	1	< 0,001	1,04	1	0,308
Momento	2952,76	6	< 0,001	1898,86	6	< 0,001
Grupo	0,04	1	0,837	0,10	1	0,754
Tempo: Momento	34,61	6	< 0,001	16,60	6	0,011
Tempo: Grupo	1,13	1	0,289	0,01	1	0,915
Momento: Grupo	29,40	6	< 0,001	16,22	6	0,013
Tempo:Momento:Grupo	2,20	6	0,900	1,84	6	0,934

*FC: Frequência Cardíaca. †Chisq: razão de verossimilhança. ‡Df: número de graus de liberdade. §Pr(>Chisq): valor-p.

## Discussão

Nos resultados encontrados corroborou-se a hipótese desta pesquisa, indicando que o uso de dispositivo de *feedback* influencia na atividade física e na redução da percepção de esforço, medida pela Escala de Borg, na realização das manobras de SBV durante a RCP, assim como a FC também é influenciada. Ao final da prática simulada, durante o *debriefing* , vários enfermeiros relataram que o dispositivo contribuiu no dimensionamento da própria força e ritmo de compressão, aplicando esforços necessários para manter a qualidade das compressões, evitar excessos e prevenir o cansaço precoce, por considerarem a atividade extenuante, principalmente aos profissionais mais sedentários.

Os achados são relevantes para identificar o nível de esforço despendido no atendimento em PCR e ponderar sobre as medidas de desempenho do profissional, ao prover o SBV com ou sem dispositivo de *feedback* , durante a RCP. Tais medidas poderão ser úteis para melhor compreender os aspectos que influenciam a *performance* e prover recomendações no delineamento de diretrizes e protocolos, para elevar a qualidade das manobras nas reanimações e a sobrevivência pós-PCR.

Nessa direção, a AHA complementou o estabelecido nas diretrizes de 2015, e em 2018 destacou, em relação às compressões torácicas externas, sobre a possibilidade de revezamento nas compressões, a cada 2 minutos – ou antes se houver cansaço – visando prevenir a fadiga e o cansaço, que comprometem a qualidade das manobras, principalmente das compressões.^6^

Para avaliar a percepção do esforço na RCP, a escala de Borg tem sido aplicada em diferentes contextos, como ferramenta de monitoração não invasiva da intensidade de esforço físico. Está relacionada com variáveis fisiológicas, como intensidade do exercício, FC e consumo de oxigênio - VO_2_. O aumento dos valores dessas variáveis é diretamente proporcional à percepção do esforço, evidenciando forte relação com a FC,^7^ corroborando o identificado no presente estudo. O estresse físico gera respostas fisiológicas, com VO_2_, ventilação, FC e concentração de lactato, cujas alterações se traduzem em sinais sensitivos que modificam a escala de Borg.^8^ É uma métrica de fácil aplicação e baixo custo; empregada em diversas áreas, inclusive esporte de alto rendimento e reabilitação. Permite monitorar alterações nos sistemas cardiorrespiratório, metabólico, neuromuscular, decorrentes do exercício físico.^9 , 10^

No contexto da reanimação, inúmeros estudos aplicaram a escala de Borg em diversos cenários. Em simulação de PCR em áreas montanhosas, a escala indicou que RCP com compressões contínuas em ambiente hipóxico,^11^ dentro da câmara hipobárica, simulando PCR em grandes altitudes, deteriora a condição do reanimador, com maior percepção do esforço e cansaço.^12^ Analogamente ocorre em RCP simulada em ambiente aeroespacial de microgravidade^13^ e no interior de veículos em movimento, mais exaustivo no interior de helicóptero do que na ambulância.^8^

A avaliação da percepção de esforço em RCP realizada em diferentes ciclos fornece informações importantes na compreensão da exaustão física e a relação com a qualidade da reanimação. Em ciclos de 30:2 e 15:2, durante dois minutos, a fadiga foi semelhante em ambos, com piora da qualidade das compressões em ciclos mais longos.^14^ A percepção de esforço foi maior e sensação de fadiga geral em RCP inclusive com compressões contínuas, em relação aos ciclos de 30:2, durante 30 minutos.^15^

Na aplicação da escala de Borg, cabe ressaltar a importância da familiarização prévia com o instrumento para que os escores indicados pelos participantes correspondam à percepção mais próxima da realidade. Por vezes, o participante expressa um valor, equivalente à menor percepção de esforço, quando na realidade, a manifestação de esforço respiratório e cansaço parece denotar correspondência a outro valor mais alto. Ciente de que está participando de uma simulação avaliativa, o efeito *Hawthorne* não pode ser descartado, ainda que os dados obtidos no registro de frequência cardíaca sejam mais objetivos.

Considerando que a qualidade das manobras de reanimação é dependente da condição física da pessoa que realiza as compressões, cansaço e fadiga são fatores que podem influenciar e por vezes comprometer, negativamente, na sobrevivência do assistido.^16^ No presente estudo, verificou-se elevação acentuada da percepção de esforço nos dois primeiros minutos, alguns profissionais quase chegaram ao nível de exaustão. Já os enfermeiros praticantes de atividades físicas regulares relataram menor cansaço durante as compressões.

O monitoramento da qualidade da RCP, em PCR intra e extra-hospitalar, ainda é um desafio. Envolve métricas tradicionais de taxa e profundidade de compressão torácica e recuo do tórax, mas também inclui parâmetros como a fração de compressão torácica, evitando ventilação excessiva, dinâmica da equipe de ressuscitação e desempenho do sistema no monitoramento da qualidade.^2^ Dentre as estratégias de monitoramento, os dispositivos de *feedback* são recursos tecnológicos que permitem o acompanhamento do desempenho na RCP, em relação a diversos parâmetros, como taxa de compressão e profundidade, fração de fluxo, frequência e volume de ventilação, entre outros e são empregados como indicadores de qualidade na análise dos atendimentos de PCR.

Existem diversos tipos de dispositivos, desde os mais simples, como metrônomos, aos mais complexos, como desfibriladores e simuladores integrados com *softwares* e sensores de pressão, para avaliação de compressões e ventilações, em treinamentos ou atendimentos em tempo real.^17^ Com o avanço da tecnologia vestível, recursos do tipo *wearable* visam evitar iatrogenias e lesão de pele durante as compressões, e facilitar o posicionamento das mãos, considerando que em cerca de dois terços das reanimações há falhas na posição.^18^ O *Smartwatch* com aplicativo fornece *feedback* audiovisual em tempo real; em simulação de RCP em ciclos de 30:2, por dois minutos, as taxas de compressão, profundidade e porcentagem de RCP de alta qualidade foram significativamente melhores no grupo intervenção.^19^ Assim como no presente estudo, a dinâmica de simulação foi similar e após a prática, os enfermeiros referiram que o dispositivo ajuda a controlar a força empregada nas compressões, reduzindo o esforço e menor percepção do cansaço.

Aplicando a tecnologia na reanimação, os dispositivos de *feedback* receberam destaque nas diretrizes AHA/2020, considerando a importância da avaliação precisa das habilidades e do *feedback* para melhorar o desempenho subsequente. Infelizmente, o desempenho inadequado da RCP é comum, ainda que seja difícil para os provedores e instrutores detectá-lo, dificultando o direcionamento apropriado e a melhora do desempenho futuro. A recomendações buscam equilibrar o benefício potencial do desempenho aprimorado da RCP com o custo do uso dos dispositivos.^2^

Dentre os desafios no manejo da PCR, enfatiza-se a educação, desde a de formação até a permanente aos profissionais; é fundamental para melhorar a retenção do aprendizado, a habilidade na prática das manobras e diminuir as barreiras para a ação dos provedores de Suporte Básico e Avançado de Vida – SBV e SAV. Na discussão sobre as inadequações dos modelos de cursos, a efetividade das ações educativas é relacionada com o modelo de aprendizagem, a abordagem aprendizagem espacial, a maior frequência na oferta de reforço em treinamentos rápidos com menor tempo de duração para prática deliberada e maestria, utilizando dispositivos de feedback de RCP.^2^

### Limitações

Utilizar um manequim em prática simulada envolve uma dinâmica de atendimento diferente da vida real, o que pode trazer diferenciação em relação aos resultados encontrados. O risco de efeito Hawthorne não pode ser descartado. Os participantes relataram dificuldade na respiração e aumento da sensação de cansaço, em razão do permanente uso de máscara, na pandemia por COVID-19, o que pode influenciar nos resultados.

## Conclusão

O dispositivo de *feedback* influenciou na percepção do esforço e na frequência cardíaca, respectivamente registradas por meio da escala de Borg e frequencímetro, durante a realização de manobras de SBV em simulação de PCR em adulto, indicando menor percepção de esforço e da FC no grupo de enfermeiros que utilizou o dispositivo, independentemente se atuantes em áreas críticas ou não críticas.
