# Supplementary material for: O Uso da Escala de Borg na Percepção do Esforço em Manobras de Reanimação Cardiopulmonar
Source: Arq Bras Cardiol. 2022 Dec 20;120(1):e20220240. [Article in Portuguese] doi: 10.36660/abc.20220240 (PMC9833294; doi:10.36660/abc.20220240)
Supplement: Translation

# Translation: The Use of the Borg Rating of Perceived Exertion Scale in Cardiopulmonary Resuscitation

**Authors:** Lucia Tobase, Heloisa Helena Ciqueto Peres, Thatiane Facholi Polastri, Sandra Helena Cardoso, Dhieizom Rodrigo Souza, Debora Gugelmin Almeida, Sergio Timerman

**Affiliations:** 1 Brazil Centro Universitário São Camilo, São Paulo, SP – Brazil; 2 Brazil Escola de Enfermagem da Universidade de São Paulo – Departamento de Orientação Profissional, São Paulo, SP – Brazil; 3 Brazil Universidade de São Paulo Instituto do Coração, São Paulo, SP – Brazil; 4 England Bournemouth University Faculty of Health and Social Sciences, Bournemouth – England

**Keywords:** Heart Arrest, Physical Exertion, Cardiopulmonary Resuscitation, Nursing Care, Emergencies

## Abstract

**Background:**

A cardiopulmonary arrest is a critical event whose survival rate is related to the quality of resuscitation maneuvers combined with the use of technology. It is important to understand the perception of fatigue during this procedure, aiming to improve the effectiveness of compressions to increase the chances of survival.

**Objectives:**

To apply the Borg rating of perceived exertion scale (Borg scale) to analyze the exertion perceived by nurses during cardiopulmonary resuscitation maneuvers using a feedback device.

**Methods:**

Experimental study with a randomized distribution of nurses in a teaching hospital. Perceived exertion during simulated cardiopulmonary resuscitation with/without a feedback device was assessed using the Borg scale. The statistical significance level of 5% was adopted.

**Results:**

69 nurses working in critical and non-critical adult care units were included. Perceived exertion and heart rate were lower in the intervention group (p<0.001), influenced by the feedback device, with no significant difference between critical and non-critical units.

**Conclusions:**

The Borg scale proved to be adequate for the proposed objectives. The feedback device contributed to lower exertion and heart rate reduction during resuscitation maneuvers. The low cost and ease of application favor its use during training and real-time resuscitation attempts to assess performance using a feedback device to reduce exertion and perception of fatigue. It allows reflection on the intervening factors and resources that can influence the quality of resuscitation attempts and the chances of survival.

## Introduction

Cardiopulmonary arrest (CPA) is responsible for approximately 17.8 million deaths/year^1^worldwide. Despite advances in the resuscitation field, since 2012, survival remains at around 8-10%. The understanding of epidemiology is still limited by the lack of global and regional data and reliable records of CPA events, particularly in low- and middle-income countries. The 2020 American Heart Association (AHA) guidelines highlight high-quality cardiopulmonary resuscitation (CPR) as a factor for successful intervention and a higher survival rate.^2^

The quality of CPR performance depends on different factors, including the rescuer’s physical condition, tiredness, and fatigue, which can compromise survival. Rotation between rescuers every 2 minutes aims to avoid fatigue and poor-quality performance. Fatigue is common after 1 minute of CPR and is usually not identified by the rescuers, even 5 minutes or more after the beginning of the intervention.^2 , 3^

The Borg rating of perceived exertion scale (Borg scale) can be applied to measure the intensity of physical activity. It is an effective tool to predict performance and define strategies to increase the quality of physical performance.^4^The measurement of recovery levels can also be analyzed using the Borg scale^5^after physical exertion. In the context of CPR, it is used to assess the quality of performance and the chances of improving resuscitation attempts.^4^

Feedback devices are encouraged during real-life resuscitation attempts and CPR training.^2^Despite clear evidence that providing high-quality CPR improves resuscitation outcomes, few healthcare organizations apply consistent strategies for monitoring the quality of CPR. Consequently, there is an unacceptable disparity in the quality of care provided and consequent survival rates, reducing the opportunity to save more lives.^6^

The present study hypothesizes differences in the perception of exertion when providing simulated CPR, with or without feedback devices. It aims to apply the Borg scale to analyze the exertion perceived by nurses during simulated CPR with a feedback device.

## Methods

### Study design

Randomized controlled trial study to compare the influence of the feedback device on the perception of exertion during simulated CPR performed by nurses, from October to November 2020. Double blinding was not feasible since the researcher herself applied the Borg scale instrument. The variables perception of exertion and heart rate (HR) were measured with a frequency meter, considered a precise sensor that provides good quality measurements. Before using the scale, permission was requested (https://borgperception.se/) and permitted after an explanation of its use for this study. The Research Ethics Committee approved the research.

### Study location

Teaching, public, general, medium-sized, and medium complexity teaching hospital, located in São Paulo, SP, Brazil.

### Study participants

Nurses working in critical (Intensive Care Unit, Emergency Room, Surgical Center, and Obstetric Center) and non-critical (Medical Clinic, Surgical Clinic and Outpatient Clinic) adult care units.

### Inclusion and exclusion criteria

Nurses from adult care units were included, and those who worked only in administrative activities or as instructors in Basic or Advanced Life Support courses were excluded. In attention to safety, professionals unable to perform or complete the activity in full due to physical limitations or pregnancy or who had symptoms of pain or health problems were also excluded.

### Outlining the methodological path

1. Presentation of the survey to service managers.
2. Recruitment: e-mail with the research information was sent to the nurses, and consent was obtained before the study began.
3. Scheduling and carrying out the practical activity in two stages: 1^st^) Baseline: verification of Basic Life Support skills. For theoretical alignment and updating of the AHA/2020 guidelines, participants accessed an online course developed by the researcher. 2^nd^) After the theoretical study, the nurses participated in a second practical activity, like the first.

According to a computer-generated random distribution list established by the statistician, participants were allocated into two groups, intervention and control. Respectively, they simulated CPA care and Basic Life Support maneuvers, with and without a feedback device. After guidance and briefing on the activity and available resources, presentation and ambiance with the scenario and the instruments to be used, the Polar H10^®^frequency meter was placed on each nurse to measure heart rate (HR). The records stored in the equipment’s internal memory were transferred via Bluetooth to cell phones and tablets, which were later accessed on the website (https://flowpolar.com) and tabulated in an *Excel* ^®^spreadsheet for information management.

The practical scenario consisted of a) presentation of the clinical case; b) nurse 1 identifies the CPA and starts chest compressions; c) nurse 2 takes over the ventilations and uses the manual defibrillator in AED mode. This continued for 2 minutes; d) pause and rest for 10 minutes in the end, hand and material hygiene, and restart the activity, with role reversal between professionals. The Borg scale was applied when the nurse performed the compressions. Two evaluators monitored each scenario. Little Anne QCPR^®^simulator manikin with smartphone viewable feedback device (QCPR instructor app) and emergency trolley provided by the hospital containing a rigid board, bag-valve-mask, flowmeter with extension and manual defibrillator in AED mode –Biphasic Defibrillator Zoll^®^model were used.

The Borg scale was previously presented to the participants for clarification of the criteria and familiarization with the tool. It was used during the practical activity, in the first and second minutes of chest compressions, and after the scenario to assess the recovery from fatigue. The HR values on the frequency meter were recorded. The values of the Borg scale (6-20) used in the present research varied as follows: 6-11 representing the minimum exertion, 12-16 for sustainable exertion and 16-20 for non-sustainable exertion until exhaustion.

### Statistical analysis

In the descriptive and inferential statistical analyses, the software R^®^4.1.0 was used, adopting a 5% significance level. Descriptive statistics were used to explore demographics. Variables of interest included the outcome related to the Borg scale perceived exertion score and heart rate variation during CPR. Categorical variables, such as participants’ performance in critical and non-critical units, were described in relative and absolute frequencies. Skewness, Kurtosis and Shapiro-Wilk tests were used to determine normality. Continuous variables showed normal distribution and were described through mean and standard deviation (SD). A mixed effects model was used to compare perceived exertion and HR variables.

## Results

Of the 190 nurses at the institution, 72 (38%) were excluded for working in areas unrelated to the study’s focus. Of the 118 (62%) eligible, 62 (53%) worked in critical units and 56 (47%) in non-critical units. Of these, 49 (41%) were excluded due to health problems, sick leave, remote work due to the COVID-19 pandemic and dismissal from the institution, as shown in Figure 1 .

**Figure 1:** Flowchart of recruitment, allocation, and analysis of participating groups. Source: author (adapted from the CONSORT flowchart).

Sixty-nine nurses were included, 35 (51%) from a critical unit and 34 (49%) from a non-critical unit; three (04%) concluded a doctorate, 15 (22%) a master’s degree, 44 (64%) a specialization and seven (10%) did not have a postgraduate degree. Other classifications of the participants’ profiles are shown in Table 1 .

**Table 1:** Profile of participants, distributed between control and intervention groups

Variables	Intervention	Control
	
	n=36 (52%)	n= 33 (48%)
Gender	04 (11%) men	03 (09%) men
	32 (89%) women	30 (91%) women
Age (mean in years/standard deviation)	41.6 (10.64)	41.4 (10.16)
Body mass index (mean/standard deviation)	25.7 (4.38)	25.6 (4.25)
Critical unit	20 (55%)	15 (45%)
Non-critical unit	16 (44%)	18 (55%)
Professional training (average in years/standard deviation)	17.6 (10.31)	17.5 (9.95)
**Professional working time:**		
At the institution (average in years/standard deviation)	15.7 (10.02)	15.5 (9.88)
In the unit (average in years/ standard deviation)	12.6 (9.25)	12.5 (9.27)
Practice of regular physical activity	15 (41.6%)	11 (33.3%)

In the second stage of the study, indicated as post-time, the quantitative descriptions of the values are related to the measurements verified through the Borg scale and the frequency meter during the two minutes of CPR and in the recovery period in both the intervention and control groups. For the perception of exertion, it was found that initially, the average of 6 indicated an absence of tiredness when starting the practical activity. Progressively, at the end of the first minute of CPR, equivalent to five cycles of 30 compressions alternated with two ventilations, the score between 13-14 indicated the perception of moderate fatigue in the tolerable exertion of the activity. At the end of the second minute, after completing about 10 cycles alternating 30 compressions and two ventilations, the score variation 14 indicated moderate exertion, approaching the limit for score 15 in the perception of high intensity and difficulty in performing the activity. In this direction, there was a progressive increase in HR recorded from the initial activity period and during the cycles of 30 compressions and two ventilations.

Conversely, in the recovery stage, there was a decrease in the values on the Borg and HR scales, indicating that after four minutes, the perceived exertion score and HR measure approached the values verified at the beginning of the activity, as demonstrated in Table 2 .

**Table 2:** Borg scale and HR in the control and intervention groups in the first and second minutes of CPR and during recovery

			BORG		HR*	
	
Group	Time	n	Average	SD^**†**^	Average	SD
**Control**	Start	33	6.00	0.00	86.50	10.80
	1 minute	33	14.00	2.15	121.00	16.00
	2 minutes	33	14.90	2.02	123.00	16.80
	1-minute recovery	33	11.50	1.44	94.20	16.20
	2-minute recovery	33	9.21	1.80	82.20	13.50
	3-minute recovery	33	7.88	1.85	79.80	12.10
	4-minute recovery	33	6.79	1.32	78.90	12.30
**Intervention**	Start	36	6.00	0.00	88.00	11.30
	1 minute	36	13.50	2.23	119.00	17.20
	2 minutes	36	14.20	2.41	121.00	18.00
	1-minute recovery	36	12.00	2.20	95.60	12.40
	2-minute recovery	36	9.97	1.98	85.50	12.00
	3-minute recovery	36	8.46	1.90	83.20	11.60
	4-minute recovery	36	6.94	1.37	81.60	10.30

* HR: Heart Rate. † SD: Standard Deviation

Regarding nurses working in critical and non-critical units, when analyzing the Borg scale scores in the first and second minutes of CPR, the values varied between 11-13, approaching 14 during CPR performance, indicating an increase in the perception of light to moderate exertion, approaching intense exertion by the end of the second minute. In the recovery period, the decrease in values at the end of the fourth minute was similar in both groups. The means were very similar, suggesting that there was no difference in the perception of exertion and the variation of HR.

In the comparative analysis between the variables (perceived exertion and HR), respectively measured through the Borg scale and the frequency meter, the mixed effects model was used, incorporating fixed and random effects simultaneously. Fixed effects are those that do not have variability, such as allocation to a control/intervention group, gender, or the age of a subject (i.e., the vast majority of variables), while random effects are subjects and variability in selection. In the context of longitudinal data, it typically corresponds to the subject being evaluated or when several judges are evaluating a set of observations, and these judges are chosen from a larger group.

The first stage or baseline was called the ‘pre’ phase, and the second stage, with randomization of the participants, with or without using the feedback device, was called the ‘post’ phase. For both variables, the results showed evidence of moment*group interaction, which indicates that the groups probably do not have the same development from the beginning of the procedure to the end of recovery. The control group showed increased perceived exertion, HR, and longer recovery. The intervention group showed lower perceived exertion, HR, and faster recovery, with a significant difference (p<0.001) in performing CPR with a feedback device, as shown in Table 3 .

**Table 3:** Borg scale and HR variation concerning time, moment, and allocation group of participants

	BORG			HR*		
	
	Chisq^**†**^	Df^**‡**^	Pr(>Chisq)^**§**^	Chisq	Df	Pr(>Chisq)
(Intercept)	4595.82	1	< 0.001	5190.58	1	< 0.001
Time	12.39	1	< 0.001	1.04	1	0.308
Moment	2952.76	6	< 0.001	1898.86	6	< 0.001
Group	0.04	1	0.837	0.10	1	0.754
Time: Moment	34.61	6	< 0.001	16.60	6	0.011
Time: Group	1.13	1	0.289	0.01	1	0.915
Moment: Group	29.40	6	< 0.001	16.22	6	0.013
Time: Moment: Group	2.20	6	0.900	1.84	6	0.934

* HR: Heart Rate.^*†*^Chisq: likelihood ratio.^*‡*^Df: number of degrees of freedom.^*§*^Pr(>Chisq): p-value.

## Discussion

The results from this study supported the hypothesis that using a feedback device influences physical activity with reduced perceived exertion, measured by the Borg Scale, in the performance of BLS maneuvers during CPR. Additionally, HR is also influenced by the use of a feedback device. At the end of the simulated practice, during the debriefing, several nurses reported that the device contributed to the perception of their own compression strength and rhythm. It enabled them to apply the necessary exertion to maintain the quality of compressions, avoid excesses and prevent early fatigue, as they considered the activity strenuous, especially for more sedentary professionals.

The findings are relevant to identifying the level of exertion spent in CPA care and considering the measures of professional performance when providing the BLS with or without a feedback device during CPR. Such measures may be useful to understand better the aspects that influence performance and provide recommendations in the design of guidelines and protocols to improve the quality of maneuvers in resuscitation and post-CPA survival.

In this direction, the AHA complemented what was established in the 2015 guidelines and highlighted in 2018 the possibility of alternating rescuers every 2 minutes - or sooner if there is fatigue – during CPR performance. This is in an attempt to prevent fatigue, which can compromise the quality of CPA care maneuvers, especially chest compressions.^6^

To assess perceived exertion in CPR, the Borg scale has been applied in different contexts as a tool for non-invasive monitoring of physical exertion intensity. It is related to physiological variables, such as exercise intensity, HR, and oxygen consumption - VO_2_. The increase in the values of these variables is directly proportional to the perception of exertion, showing a strong relationship with HR,^7^supporting what was identified in the present study. Physical stress generates physiological responses, with VO_2_, ventilation, HR and lactate concentration, whose changes translate into sensitive signals that modify the Borg scale.^8^It is an easy-to-apply and low-cost tool; used in various areas, including high-performance sports and rehabilitation. It allows monitoring changes in the cardiorespiratory, metabolic, and neuromuscular systems resulting from physical exercise,^9 , 10^

In the context of resuscitation, several studies used the Borg scale in different scenarios. In two CPR simulation studies conducted in mountainous areas, the Borg scale indicated that CPR with continuous chest compressions in a hypoxic environment,^11^inside a hypobaric chamber, simulating CPR at high altitudes, deteriorates the rescuer’s condition, with a greater perception of exertion and fatigue.^12^Similar results are found in simulated CPR conducted in a microgravity aerospace environment^13^or inside moving vehicles, with more exhaustion perceived inside a helicopter than in an ambulance.^8^

The assessment of perceived exertion during CPR performed in different compression: and ventilation cycles provides important information in understanding physical exhaustion and its relationship with the quality of resuscitation attempts. During two minutes of CPR using 30:2 and 15:2 cycles, fatigue was similar in both, with worsening quality of compressions in longer cycles.^14^However, the perception of exertion and the feeling of general fatigue during CPR performance was greater when 30:2 cycles were used for 30 minutes.^15^

It is worth highlighting the importance of prior familiarization with the tool so that the scores indicated by the participants using the Borg scale correspond to the closest perception of reality. Sometimes, participants may express a value equivalent to the lowest perception of exertion when in reality, the manifestation of respiratory exertion and fatigue seems to represent a higher value in the Borg scale. This may be explained by the Hawthorne effect, as participants know they were participating in an evaluative simulation study. Therefore, this phenomenon cannot be ruled out, even though the data obtained from HR recordings are more objective.

Considering that the quality of resuscitation maneuvers depends on the physical condition of the person performing CPR, tiredness and fatigue are factors that can influence and sometimes negatively compromise the survival of the assisted person.^16^In the present study, there was a marked increase in perceived exertion in the first two minutes, with some professionals almost reaching the level of exhaustion. Not surprisingly, nurses who practice regular physical activities reported less fatigue during chest compressions.

Monitoring CPR quality for in-hospital and out-of-hospital cardiac arrest is still a challenge. It involves traditional metrics such as chest compression rate, depth, and chest recoil but also includes parameters such as chest compression fraction, avoiding excessive ventilation, dynamics of the resuscitation team, and system performance for quality monitoring.^2^Among the monitoring strategies, feedback devices are technological resources that allow assessment and quality indicators of CPR performance concerning several metrics, including compression rate, depth, flow fraction, ventilation frequency and volume.

There are several types of feedback devices, from the simplest, such as metronomes, to the most complex, such as defibrillators and simulators integrated with software and pressure sensors, for evaluation of compressions and ventilations.^17^With the advancement of technology, wearable devices aim to avoid iatrogenic and skin injuries during chest compressions and facilitate hand positioning, potentially improving this metric. This is an important function considering that about two-thirds of resuscitation attempts present failures in the hand position.^18^Smartwatch with CPR-related apps, for example, provides real-time audio-visual feedback during CPR performance. A study from Cheng et al. demonstrated that during simulated CPR using the 30:2 cycles for two minutes, compression rate, depth and percentage of high-quality CPR were significantly better in the intervention group.^19^As in the present study, CPR performance was similar in the intervention and control groups; however, after the scenario, the nurses reported that the device helped to control the force used during compressions, reducing the exertion and lessening the perception of fatigue.

Feedback devices have been highlighted in the AHA 2020 guidelines, considering the importance of accurate assessment of skills and feedback to improve subsequent performance. Unfortunately, poor CPR skills are still common, and it is challenging for providers and instructors to detect poor quality performance, making it difficult to target and improve future performance properly. Finding the balance between the potential benefit of improved CPR performance and the cost of investing in wearable devices is still recommended.^2^

Among the challenges in managing CPA, education is also emphasized, from training to the retention of CPR skills. It is essential to improve learning, maximize skill retention and reduce barriers to initiating Basic and Advanced Life Support. Evidence about the inadequacies of the training models suggests that the effectiveness of the educational actions, such as CPR knowledge and skills, is related to the training model. Different approaches have been suggested, including spaced learning, distributed or low-dose high-frequency practice, and mastery learning using feedback devices to reinforce knowledge and skills during CPR performance.^2^

### Limitations

Using a mannequin in simulated practice involves different care dynamics from real-life performance, which could affect the results. The risk of the Hawthorne effect cannot be ruled out. Participants reported difficulty in breathing and increased feelings of fatigue due to the permanent use of a mask during the COVID-19 pandemic, which could have influenced the results.

## Conclusion

The feedback device influenced the perception of exertion and heart rate, respectively recorded using the Borg scale and frequency meter, during the performance of BLS maneuvers in adult CPA simulation. The results indicated a lower perception of exertion and HR in the group of nurses who used the device, whether working in critical or non-critical areas.
